# ^18^F-FDG PET/CT Associates With Disease Activity and Clinical Recurrence of AOSD Patients

**DOI:** 10.3389/fmed.2021.668323

**Published:** 2021-05-11

**Authors:** Xian Li, Chuning Dong, Xiaowei Ma, Yunhua Wang

**Affiliations:** Department of Nuclear Medicine, The Second Xiangya Hospital, Central South University, Changsha, China

**Keywords:** ^18^F-FDG, AOSD, disease activity, recurrence, PET/CT, still's disease

## Abstract

**Objective:** The purpose of this study was to explore the value of ^18^F-FDG PET/CT in monitoring the disease activity and predicting the prognosis of the Adult-onset Still's disease (AOSD).

**Methods:** We retrospectively analyzed the electronic medical records of 45 AOSD patients who underwent ^18^F-FDG PET/CT in the Second Xiangya Hospital. PET/CT imaging and clinical information were retrospectively reviewed and analyzed. ^18^F-FDG uptake was assessed by measuring standard uptake value (SUV) in the spleen, liver, bone marrow, and lymph nodes. The spleen-to-liver ratio of the SUVmax (SLRmax) and SUVmean (SLRmean), the bone-to-liver ratio of the SUVmax (BLRmax), and SUVmean (BLRmean), and the lymph nodes-to-liver ratio of the SUVmax (LyLRmax) were calculated. Clinical and laboratory information were collected and evaluated for association with metabolic parameters of ^18^F-FDG PET/CT. The influencing factors for recurrence within 1 year were analyzed to determine whether ^18^F-FDG PET/CT can predict the prognosis of AOSD patients.

**Results:** Elevated ^18^F-FDG uptake could be observed in bone marrow, spleen, and lymph nodes of AOSD patients. Correlation analysis between ^18^F-FDG uptake of organs and laboratory examinations showed that SLRmean positively correlated with LDH, AST, ferritin, and the systemic score (*r* = 0.572, 0.353, 0.586, and 0.424, *P* < 0.05). The SLRmean had the highest correlation with ferritin (*r* = 0586, *P* < 0.001). All metabolic parameters in spleen, including SUVmax, SUVmean, SLRmax, and SLRmean, are positively correlated with LDH level (*r* = 0.405, 0.539, 0.481, and 0.572, *P* < 0.05). Bone marrow SUVmax, BLRmax, and BLRmean were correlated with C-reactive protein (CRP) level (*r* = 0.395, 0.437, and 0.469, *P* < 0.05). Analysis of the influencing factors of recurrence within 1 year showed that the spleen SUVmax, spleen SUVmean, SLRmax, SLRmean, ferritin, and the systemic score of the recurrence group was significantly higher than the non-recurrence group (*P* < 0.05). The SLRmean cutoff of 1.66 with a sensitivity of 72.7% and specificity of 80.0% had the highest performance in predicting recurrence.

**Conclusion:** The glucose metabolism of the liver, spleen, and bone marrow of AOSD patients were correlated with laboratory inflammatory indicators and system score, suggesting that ^18^F-FDG PET/CT could be applied to evaluate disease activity. Moreover, spleen ^18^F-FDG uptake may be a potential biomarker for predicting clinical prognosis of AOSD patients.

## Introduction

AOSD is a multisystemic autoinflammation disorder usually affecting young adults, which is associated with many inflammatory factors including IL-1 (inetleukin-1, IL-1), IL-6, IL-8, tumor necrosis factor alpha (TNF-α), and interferon gamma (IFN-γ) (1, 2). AOSD is characterized by four cardinal symptoms, fever, rash, arthralgia, and increased leukocyte and neutrophil counts. Many other manifestations can also occur, such as odynophagia, myalgia, myositis, lymphadenopathy, and splenomegaly. However, nonspecific clinical sign or biological abnormality is unable to ascertain the diagnosis of AOSD. The most widely used diagnosis criteria of AOSD is Yamaguchi criteria, but the criteria ought to exclude infections, malignant tumors, and other rheumatic diseases. Then performing a comprehensive diagnostic work-up is challenging in clinical practice.

Accompanied with activation of systemic inflammation, AOSD is closely related to inflammatory activity and may develop severe and life-threatening complications, such as macrophage syndrome, disseminated intravascular coagulopathy (DIC), thrombotic thrombocytopenic purpura (TTP) (3–5). The most common methods for assessing AOSD activity are systemic score and laboratory tests, such as erythrocyte sedimentation rate (ESR), C-reactive protein (CRP), lactate dehydrogenase (LDH), aspartate aminotransferase (AST), alanine aminotransferase (ALT), and plasma ferritin (6, 7). ^18^F-FDG PET/CT, which reflect the glucose metabolism of organs, can be used in differential diagnosis of AOSD and malignant tumors and other autoimmune diseases (8, 9). However, ^18^F-FDG PET/CT imaging for assessing AOSD disease activity have not been well-established. Some researches have demonstrated ^18^F-FDG PET/CT may be associated with the activity of the disease (10, 11), but their limitation is a small sample sized. Thus, more investigations are required to certify its value for accessing disease activity. Besides, a reliable method for predicting the therapeutic response and outcome has not been established (12). Therefore, it is critically important to develop new tools for effectively monitoring disease activity and predicting the outcome of AOSD in order to prevent fatal complications.

In this study, we retrospectively investigated a group of patients with AOSD to explore the role of ^18^F-FDG PET/CT imaging in evaluating the disease activity and predicting prognosis.

## Materials and Methods

### Patient Selection

The medical records and PET/CT images of 45 AOSD patients at the Second Xiangya Hospital, Central South University, from January 2015 to June 2019 were reviewed. Inclusion criteria: (1) Met the Japan's Yamaguehi criteria (13), it contains four major criteria (fever, rash, arthritis, and leukocytosis) and five minor criteria (sore throat, lymphadenopathy, hepatomegaly/splenomegaly, altered liver function test, and negative for antinuclear antibodies and rheumatoid arthritis) criteria. A total of five or more criteria are required to make the diagnosis, including at least two major criteria; (2) Followed up by telephone or outpatient service for more than 6 months; (3) Complete laboratory examinations within 3 days before and after ^18^F-FDG PET/CT examination; (4) No previous history of malignant tumor; (5) Complete clinical data. Exclusion criteria: (1) previous history of tumors; (2) patients who had progressed to other rheumatic diseases or malignant tumors; (3) Incomplete clinical data. This retrospective study was approved by Medical Ethical Committee of The Second Xiangya Hospital.

### Clinical Information and Laboratory Data and Therapeutic Data Collection

Clinical information included age, sex, and length of disease. Laboratory data included cell counts of white blood cell, neutrophil, lymphocyte, monocyte, and platelet and hemoglobin, ALT, AST, ESR, CRP, LDH, and ferritin levels. All laboratory tests were measured within 3 days of the date of the PET/CT scan. Other tests included bone marrow aspiration and lymph node biopsy. Therapeutic Data included drug category (glucocorticoids or nonsteroidal anti-inflammatory drugs or antirheumatic drugs), medication time, sequence of drug therapy and PET/CT examination.

The systemic score standard is mainly based on clinical manifestation scores for disease activity evaluation (14), with a score of 0–12, and 1 point for each of the following clinical manifestations: fever, typical rash, pleurisy, pneumonia, pericarditis, hepatomegaly, or abnormal liver function, splenomegaly, large lymph nodes, sore throat, myalgia, abdominal pain, and white blood cell count ≥15 × 10^9^/L. The total score ≥ 2 points is regarded as an active stage of AOSD.

### Criteria for Recurrence

All patients were followed up for at least 6 months to exclude other rheumatic diseases or malignant tumors. Recurrence refers to the disappearance of clinical manifestations such as fever and rash after systemic treatment and the discharge of related laboratory indicators to the normal range. During the one-year follow-up period after discharge from the hospital, under the premise of taking regular medication and eliminating other pathogenic factors such as infection, patients recurred fever, rash, arthralgia, sore throat, lymphadenopathy, splenomegaly and other AOSD-related clinical symptoms, accompanied by relevant laboratory indicators and abnormalities in imaging examinations (X-ray, CT or MRI).

### ^18^F-FDG PET/CT Image Acquisition

All ^18^F-FDG PET/CT images were acquired with a standard protocol on a dedicated PET/CT scanner (Biography mCT, Siemens Medical Systems, Germany). Patients fasted for 6 h before the PET/CT scan, and a blood glucose level below 140 mg/dL was confirmed. The PET/CT scan was performed 60 min after the intravenous administration of 5.0 MBq/Kg body weight of ^18^F-FDG. The first procedure of scanning is CT scan (120 kV, 200 mA, and layer thickness 3.0 mm). Then the PET scan was performed with an acquisition time of 1.5 min per bed position in 3-dimensional mode. Imaging ranging from the base of the skull to the middle of the thighs was acquired for each patient, then the image is reconstructed by the iterative method, and the data is transferred to the MMWP image post-processing workstation.

### PET/CT Imaging Analysis

**Figure 1:**
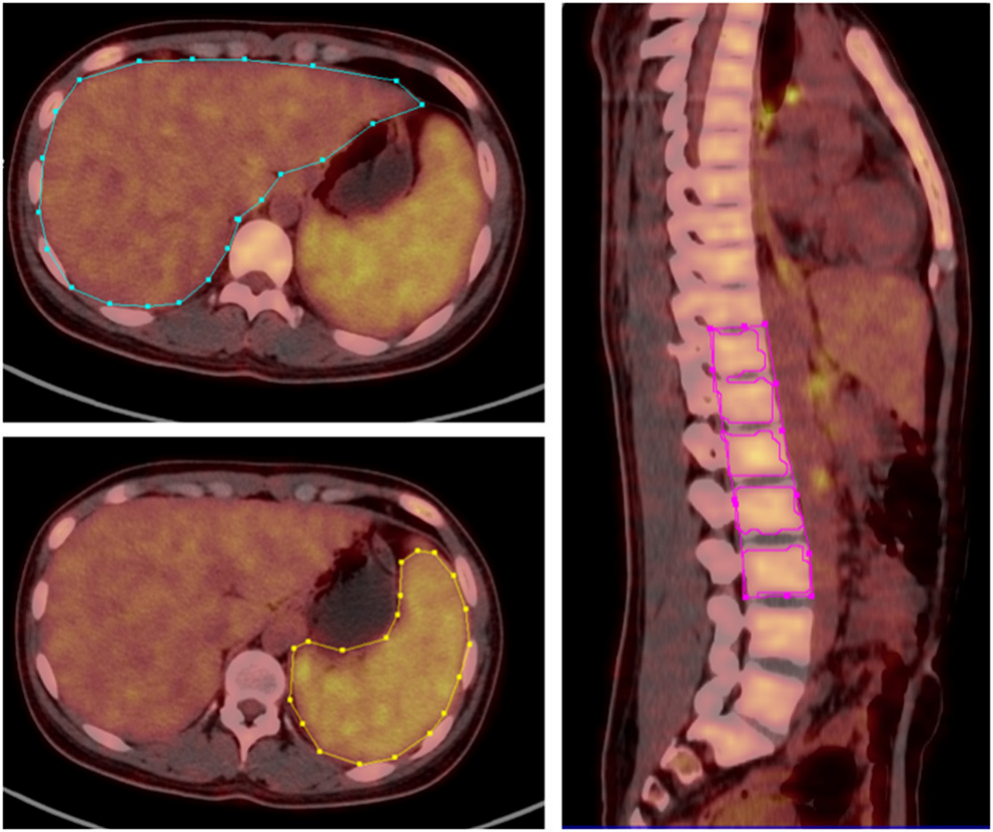
3D ROI was manually delineated according to the contours of liver, spleen and spine, SUVmax, and SUVmean of corresponding regions was obtained.

PET/CT imaging was firstly visually analyzed by two experienced nuclear medicine physicians to determine whether there was abnormal ^18^F-FDG uptake and/or structural changes in the imaging. Secondly, the location, number, ^18^F-FDG uptake, and systemic distribution of abnormal lesions were recorded. Thirdly, the maximum standardized uptake value (SUVmax) and mean standardized uptake value (SUVmean) of the liver, spleen, and bone marrow was measured and recorded by a fixed physician, using the three-dimensional region of interest (3D ROI) technique manually. The ROIs on liver, spleen, and spine were manually delineated according to the contours of target organs slice-by-slice and then reconstructed to three-dimensional region of interest (3D ROI). The bone marrow SUV measurement is obtained by delineating the ROI of five adjacent vertebral bodies (from T11-L3 spine, except for compression fractures or severe osteoarthritis changes, or those undergoing surgery due to spinal diseases Patient) (Figure 1). When an abnormal lymph node was found, the SUVmax was measured and recorded. The spleen-to-liver ratio of the SUVmax (SLRmax) and SUVmean (SLRmean) was calculated by dividing the spleen SUVmax and SUVmean by the liver SUVmax and liver SUVmean, respectively, and the bone marrow-to-liver ratio of the SUV max (BLRmax) and SUVmean (BLRmean) was calculated by dividing the bone marrow SUVmax and SUVmean by the liver SUVmax and liver SUVmean, respectively. The lymph node-to-liver ratio of SUVmax (LyLRmax) was calculated by dividing lymph node SUVmax by the liver SUVmax.

### Statistical Analysis

Data were analyzed using SPSS, version 25 (SPSS Inc.). Continuous variables are expressed as mean with SD, and continuous variables data were compared using the Student *t*-test. Categoric variables are expressed as frequencies and percentages, compared using the *x*^2^ test. Correlations between laboratory variables and metabolic parameters (SUVmax, SUVmean, SLRmean, SLRmax, BLRmean, BLRmax, and LyLRmax) were calculated by the Pearson correlation analysis. The discriminative ability of spleen SUVmax, spleen SUVmean, SLRmax, SLRmean for predicting recurrence was analyzed using area under the receiver-operating-characteristic (ROC) curve. The area under the ROC curve was presented with 95% confidence interval (CI), and the Youden index was used to identify the maximal cutoff. When *P* value was less than 0.05, the difference was considered statistically significant.

## Results

### Baseline Characteristics of Patients and Pathology and Therapeutic Data

Thirty-one female and 14 male patients with a mean age of 36.4 years (range, 16–74 years) took part in the present study. The duration of the disease ranged from 1 week to 2 years, with a mean of 109 days. The laboratory data and systemic score of 45 patients are shown in Table 1. Twenty-seven patients underwent bone marrow biopsy, all of which were myelohyperplasia. Nineteen patients underwent lymph node biopsy, of which 2 cases showed necrotizing lymphadenitis, 1 case had lymph node structural disorder, and the rest had lymph node reactive hyperplasia. Nine skin biopsies were performed, and there were no apparent abnormalities.

**Table 1:** Baseline characteristics of AOSD patients.

**Variable**	***n***	**Minimum**	**Maximum**	***x* ± *s***
WBC count (/μL)	45	2.9	31.3	13.7 ± 6.4
Neutrophil count (/μL)	45	2.1	29.3	11.4 ± 6.2
Lymphocyte count (/μL)	45	0.4	5.8	1.5 ± 1.0
Monocyte count (/μL)	45	0.1	0.9	0.5 ± 0.2
Hemoglobin (g/dL)	45	49.0	127.0	96.0 ± 18.4
Platelet count (1,000/μL)	45	81.0	531.0	256.2 ± 113.9
ESR (mm/h)	35	7.0	274.0	74.1 ± 48.4
CRP (mg/L)	36	0.8	215.0	70.5 ± 51.2
LDH(IU/L)	33	210.0	1863.8	655.1 ± 404.6
Ferritin (ng/mL)	32	2005.4	40000.0	22120.5 ± 15390.3
ALT (IU/L)	38	9.6	298.1	50.9 ± 59.9
AST (IU/L)	38	18.6	142.9	59.8 ± 34.3
Systemic score	45	2.0	10.0	6.3 ± 1.4

*WBC, white blood cell; ESR, erythrocyte sedimentation rate; CRP, C-reactive protein; AST, aspartate aminotransferase; ALT, alanine aminotransferase; LDH, lactate dehydrogenase*.

Of the 45 patients, 17 patients did not receive drug therapy before PET/CT examination, and 28 patients received medical treatment within 3 days before PET/CT examination, of which 19 patients received monotherapy (glucocorticoids/nonsteroidal anti-inflammatory drugs/conventional synthetic DMARDs) and 9 patients received combination therapy. Among all treated patients, the mean duration of treatment was 7 days, the shortest treatment time was 2 days and the longest was 30 days.

During the follow-up period, 41 patients were followed up for more than 1 year. Of those 41 patients, 37 patients received glucocorticoids as first-line treatment, 20 of whom received non-steroidal anti-inflammatory drugs simultaneously, and 17 of whom received conventional synthetic DMARDs. Two patients received single nonsteroidal anti-inflammatory drugs and anti-rheumatic drugs as first-line treatment, respectively. The average treatment time for all patients was 8.2 months, the shortest was 3 months, and the longest was 25.3 months.

### Characteristics of ^18^F-FDG PET/CT Imaging in AOSD Patients

**Figure 2:**
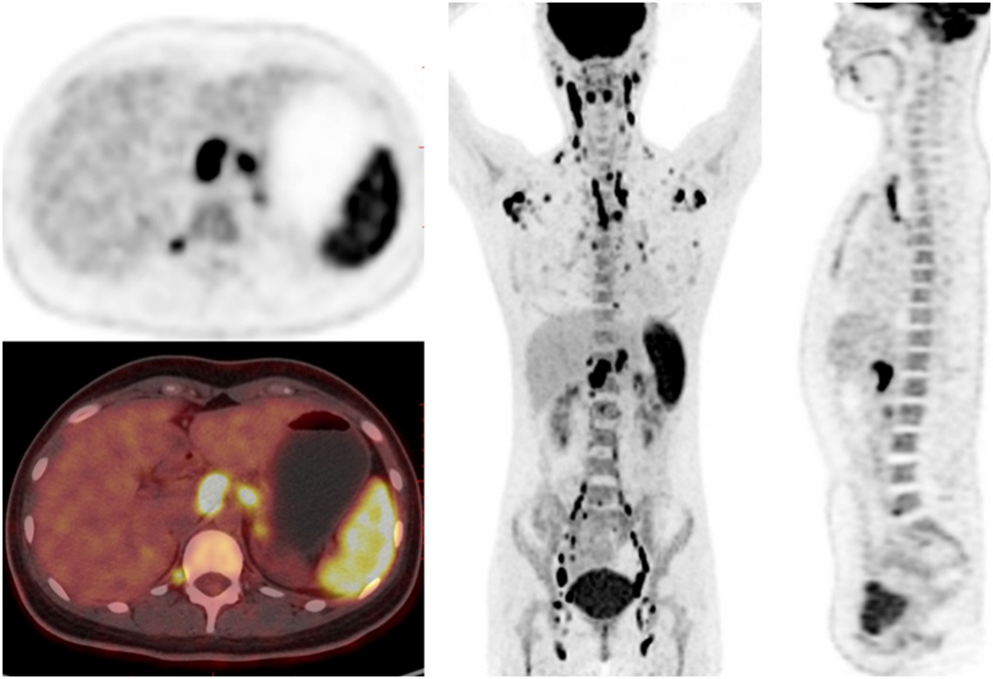
^18^F-FDG PET/CT images in a 23-year-old female of AOSD patient. PET/CT showed diffusely increased ^18^F-FDG uptake in spleen, bone marrow and lymph nodes, and reactive hyperplastic lymph nodes symmetrically distributed in the whole body.

In AOSD patients, the main manifestations of PET/CT images are diffusely increased FDG uptake in the spleen, bone marrow, and lymph nodes (Table 2 and Figure 2). The spleen glucose metabolism increased in 42 patients, all accompanied by splenomegaly. Reactive hyperplastic lymph nodes with increased ^18^F-FDG uptake of 42 patients were distributed in the whole body, most of them located in the neck and axilla. All lymph nodes were regular and oval; there was almost no fusion or calcification, and only 1 case had a tendency of fusion. Seven patients had increased FDG uptake in the joints, mainly involving the shoulder and hip joints, with joint pain in all cases. In addition, 10 patients had pleural effusion, and 2 patients had pericardial effusion.

**Table 2:** ^18^F-FDG uptakes in AOSD patients.

**Lesion**	***n***	**SUVmax**		**SUVmean**	
		**Range**	***x* ± *s***	**Range**	***x* ± *s***
Spleen	42 (93.3%)	2.28–10.52	4.82 ± 1.64	1.07–6.30	2.73 ± 1.05
Bone marrow	41 (91.1%)	2.46–10.81	5.66 ± 1.90	1.12–4.87	2.38 ± 0.83
Lymph node	42 (93.3%)	2.33–42.67	10.32 ± 7.68	–	–
Joint	7 (15.6%)	3.30–6.10	4.3 ± 0.68	–	–
Parotid gland/Salivary gland	3 (6.7%)	2.60–4.50	3.70 ± 0.98	–	–

### Association of Laboratory Variables With Metabolic Parameters

**Figure 3:**
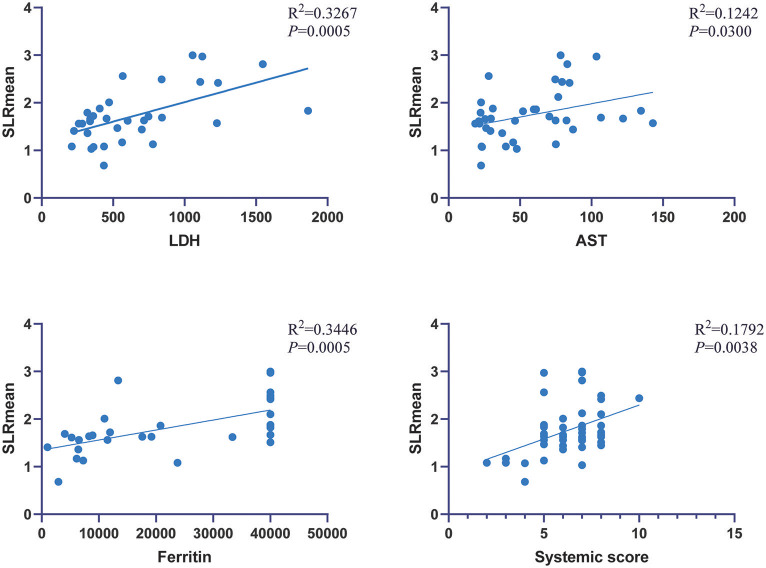
SLRmean correlated positively with LDH, AST, ferritin, and systemic score (*P* < 0.05).

**Figure 4:**
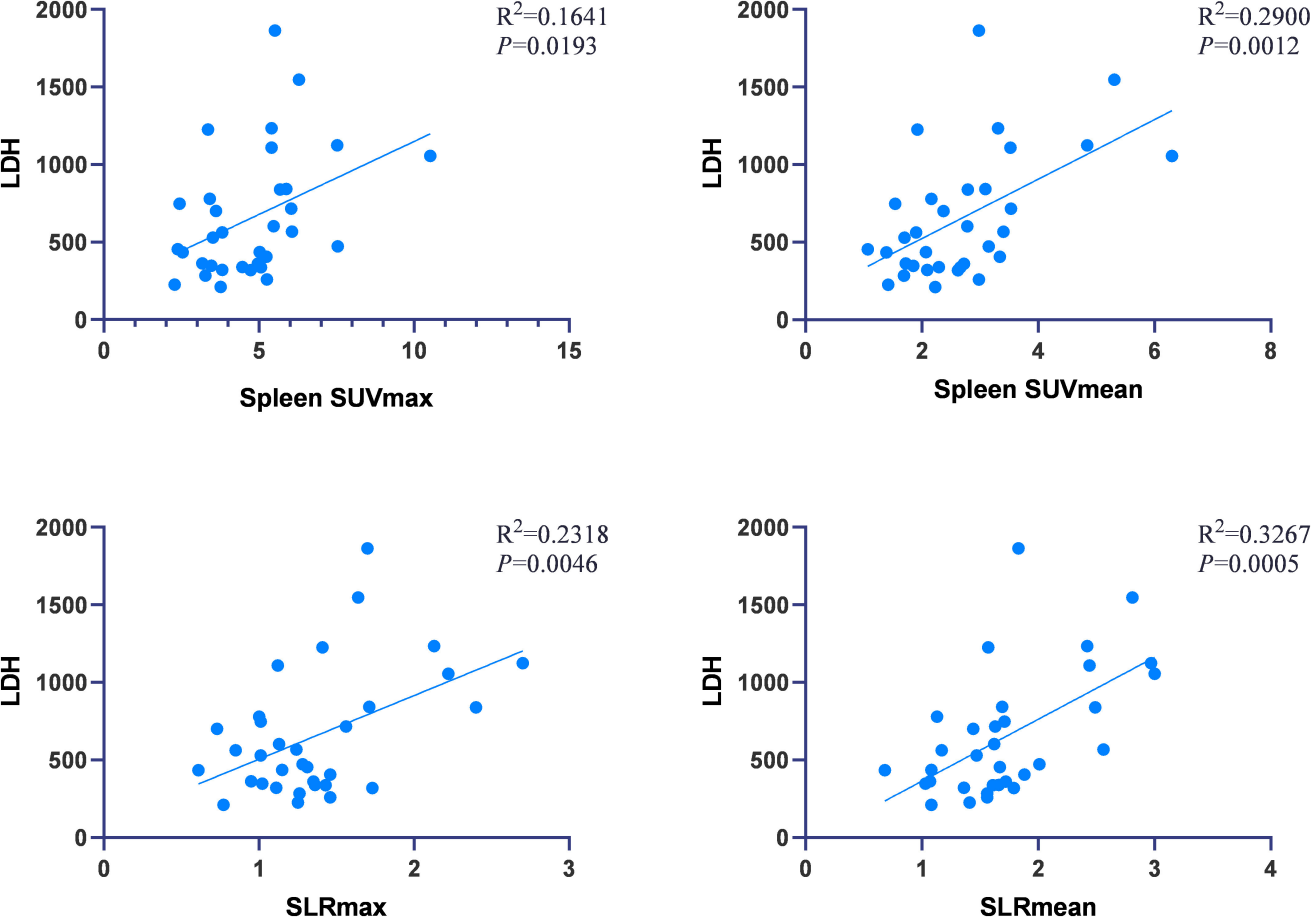
LDH was positively correlated with spleen SUVmax, spleen SUVmean, SLRmax, SLRmean, and LDH (*P* < 0.05).

In AOSD patients, correlation analysis between metabolic parameters and laboratory examination showed that SLRmean correlated positively with LDH, AST, ferritin, and the systemic score (*r* = 0.572, 0.353, 0.586, and 0.424, *P* < 0.05; Figure 3), and SLRmean had the highest correlation with ferritin (*r* = 0586, *P* < 0.001). LDH was positively correlated with spleen SUVmax, spleen SUVmean, SLRmax, SLRmean (*r* = 0.405, 0.539, 0.481, 0.572, and *P* < 0.05; Figure 4). In addition, bone marrow SUVmax, BLRmax, and BLRmean were correlated with C-reactive protein (*r* = 0.395, 0.437, 0.469, and *P* < 0.05; Supplementary Table 1). We also analyzed the association of laboratory variables between LDH, liver enzyme, ferritin, and the systemic score (Supplementary Table 2). ESR, CRP, LDH, AST, ferritin, and the systemic score are related to each other.

### Differentiation Between AOSD Recurrence Group and Non-recurrence Group

**Figure 5:**
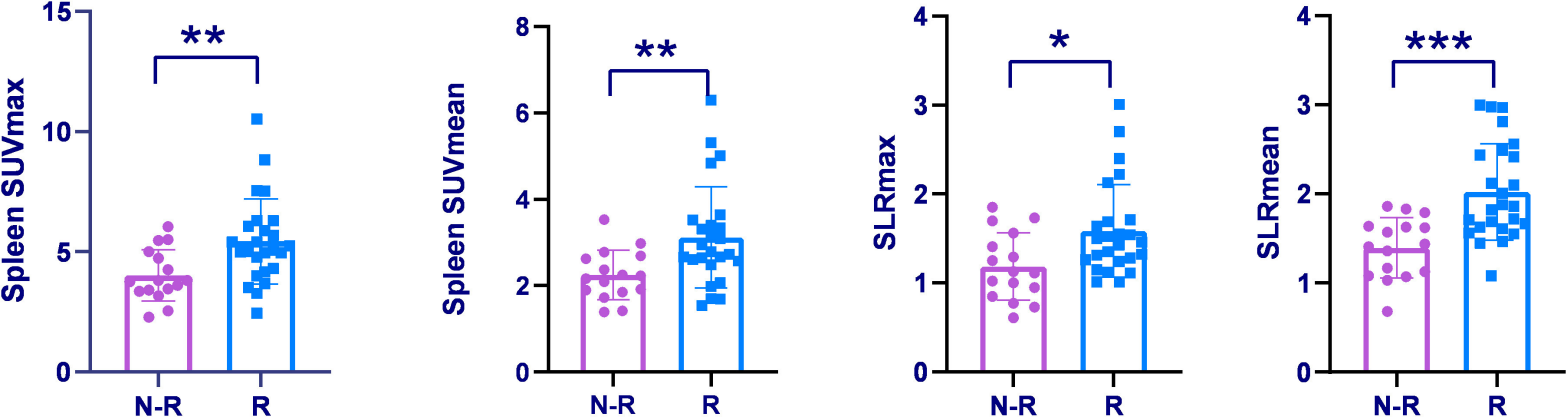
Spleen SUVmax, Spleen SUVmean, and SLRs were compared among the recurrence group and non-recurrence group. *P < 0.05, **P < 0.01, ***P < 0.001.

**Figure 6:**
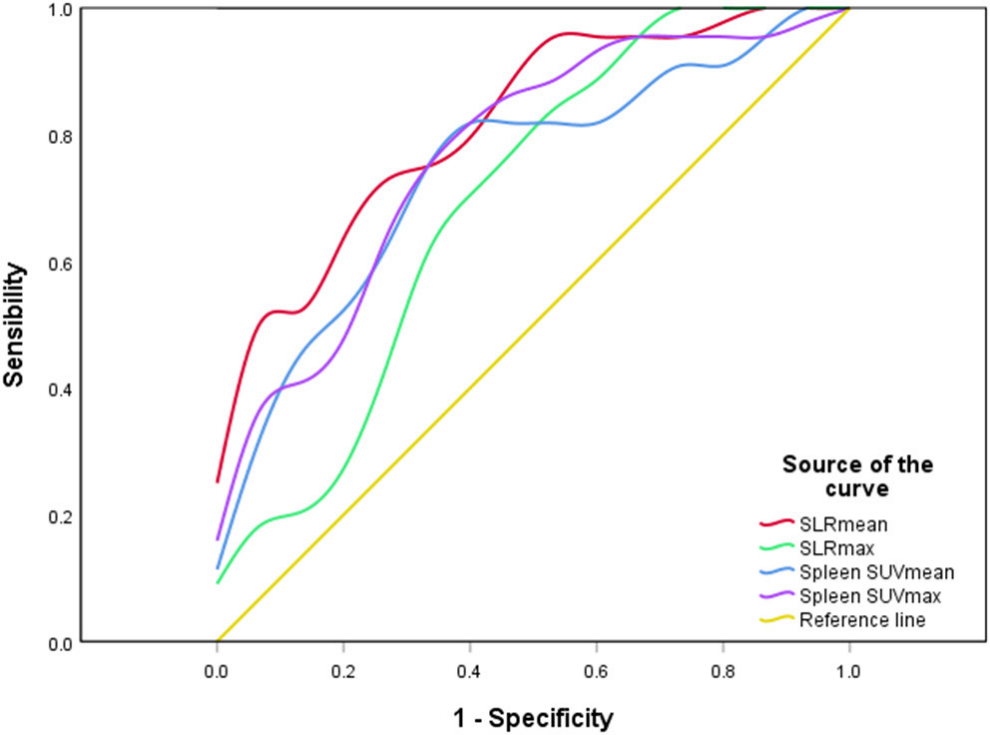
A ROC curve of spleen metabolic parameters. SLRmean had the highest ROC value (*P* < 0.001).

Forty-one AOSD patients completed more than 1 year follow-up, of which 25 patients (60.98%) relapsed, 16 patients (39.02%) did not relapse. Analysis of the influencing factors of recurrence within 1 year showed that spleen metabolic parameters (spleen SUVmax, spleen SUVmean, SLRmax, and SLRmean), ferritin, and systemic score of the recurrence group was significantly higher than the nonrecurrence group (*P* < 0.05; Table 3). ROC analysis of spleen metabolic parameters (spleen SUVmax, spleen SUVmean, SLRmax, and SLRmean), ferritin, and systemic score were performed to differentiate AOSD recurrence and nonrecurrence (Figure 5). SLRmean had the highest ROC value, and a SLRmean cutoff of 1.66 had a sensitivity of 72.7% and specificity of 80.0% (area under the ROC curve, 0.824; 95% CI, 0.692–0.957; *P* < 0.001; Table 4 and Figure 6). In addition, treatment prior to PET/CT examination did not change the metabolic parameters of the organs, there was no significant difference of metabolic parameters between the AOSD treatment group and the untreated group (Supplementary Table 3).

**Table 3:** Comparison of laboratory variables and metabolic parameters between AOSD recurrence group and non-recurrence group.

**Variable**	**Recurrence group**	**Non-recurrence group**	***P***
*n*	25 (60.98%)	16 (39.02%)	/
**Clinical variables**			
Age	37.20 ± 14.18	35.75 ± 17.08	0.770
**Sex**			
Male	4 (16.00%)	9 (56.25%)	0.018^*^
Female	21 (84.00%)	7 (43.75%)	
**Laboratory variables**			
WBC count (×10^9^/L)	14.09 ± 5.76	11.24 ± 5.90	0.133
Neutrophil count (×10^9^/L)	11.66 ± 5.89	9.27 ± 5.10	0.186
ESR^#^ (mm/h)	67.53 ± 26.50	76.14 ± 68.41	0.619
CRP^#^ (mg/L)	71.77 ± 50.87	59.59 ± 41.51	0.463
LDH^*^ (U/L)	743.30 ± 379.65	619.49 ± 450.40	0.421
Ferritin^†^ (ng/mL)	27416.00 ± 15096.67	14542.14 ± 13378.14	0.033^*^
Systemic score	6.76 ± 1.51	5.50 ± 1.59	0.015^*^
**Metabolic parameters**			
Liver SUVmax	3.54 ± 1.01	3.56 ± 0.92	0.941
Liver SUVmean	1.53 ± 0.32	1.64 ± 0.31	0.285
Spleen SUVmax	5.43 ± 1.77	4.01 ± 1.07	0.006^**^
Spleen SUVmean	3.12 ± 1.17	2.24 ± 0.57	0.008^**^
Bone SUVmax	5.91 ± 2.03	5.07 ± 1.39	0.158
Bone SUVmean	2.53 ± 0.97	2.18 ± 0.55	0.149
Lymph node SUVmax	11.48 ± 8.90	8.92 ± 6.23	0.322
SLRmax	1.59 ± 0.52	1.18 ± 0.39	0.011^*^
BLRmax	1.70 ± 0.53	1.53 ± 0.59	0.334
LLRmax	3.37 ± 2.62	2.60 ± 2.01	0.305
SLRmean	2.02 ± 0.54	1.39 ± 0.34	0.000^***^
BLRmean	1.65 ± 0.51	1.38 ± 0.47	0.107

*WBC, white blood cell; ESR, erythrocyte sedimentation rate; CRP, C-reactive protein; AST, aspartate aminotransferase; ALT, alanine aminotransferase; LDH, lactate dehydrogenase*;

# *33 patients*;

* *30 patients*;

† *28 patients*.

* *P < 0.05*,

** *P < 0.01*,

*** *P < 0.001*.

**Table 4:** Comparison of metabolic parameters and ferritin and the systemic score in differentiating between AOSD recurrence group and non-recurrence group.

**Variable**	**Cutoff**	**Sensitivity**	**Specificity**	**Area under curve**
Sleen SUVmax	4.76	72.0	75.0	0.758 (0.606–0.909)
Sleen SUVmean	2.53	80.0	69.0	0.745 (0.592–0.898)
SLRmax	1.14	84.0	56.2	0.725 (0.556–0.894)
SLRmean	1.66	72.7	80.0	0.824 (0.692–0.957)
Ferritin	36690.00	52.6	90.0	0.737 (0.547–0.927)
The systemic score	7.50	37.0	100.0	0.718 (0.533–0.904)

## Discussion

AOSD is an autoinflammation disease of unknown etiology. Its clinical symptoms are varied, typically characterized by fever, rash, arthralgia, increased leukocyte, and neutrophil counts. These manifestations and relevant laboratory tests lack specificity, so early diagnosis is problematic in clinical work. Increased ^18^F-FDG uptake involving various pathologic conditions, including malignancies, infections, and non-infectious, is greatly beneficial to the diagnosis of clinical diseases. Some studies have demonstrated the role of ^18^F-FDG PET/CT in assisting the diagnosis of ASOD (8, 9, 15). However, researches for ^18^F-FDG PET/CT evaluating AOSD disease activity are limited and whether it can assess prognosis remains unknown. In our study, we found that the glucose metabolism of the liver, spleen, and bone marrow of AOSD patients correlated with laboratory inflammatory indicators and systemic score, suggesting ^18^F-FDG PET/CT may be a useful imaging method for the assessment of disease activity throughout the body. Furthermore, our study demonstrated that ^18^F-FDG PET/CT might predict AOSD prognosis, with spleen uptake is elevated in AOSD recurrence group compared with the nonrecurrence group.

For the ^18^F-FDG PET/CT imaging of AOSD, it's common that ^18^F-FDG uptake of spleen, bone, and lymph nodes is elevated, with hepatosplenomegaly. These manifestations are consistent with previous reports (9, 11). Other lesions such as joints and glands may also accumulate a high level of ^18^F-FDG, which helps the diagnosis (16). Due to the diagnosis of AOSD is exclusive, it's necessary to rule out cancers, infections, and other autoimmune diseases firstly. Most notable is that the ^18^F-FDG PET/CT imaging of AOSD is similar to lymphoma. It's widely known that ^18^F-FDG PET/CT is essential in detecting malignant lesions. For differential diagnosis of AOSD and lymphoma, studies have shown the morphology of lymphoma's enlarged lymph nodes is variable and different, and the degree of ^18^F-FDG uptake of lymphoma is higher than AOSD (9). Moreover, ^18^F-FDG PET/CT could facilitate accurate localization of the high radioactive concentration to guide pathologic puncture to further diagnosis.

In the current study, the comparison of metabolic parameters with laboratory tests manifested distinct patterns. Metabolic parameters of the spleen (SUVmax, SUVmean, SLRmean, and SLRmax) were associated with ferritin level, liver enzymes, LDH, and systemic score, whereas bone marrow glucose metabolism was associated with CRP. Serum ferritin is an acute-phase reactant, which can elevate in various autoimmune and autoinflammation diseases, such as AOSD and systemic lupus erythematosus. An increase of more than five times of ferritin has a high specificity in the diagnosis of AOSD, and it is closely related to the activity state of the disease (6, 17). AOSD can affect a variety of lesions, of which the liver is a commonly involved organ. The mechanisms are not yet clear but may be related to the continuous activation of macrophages and the production of cytokines (18). The primary manifestation of liver damage is transaminase elevate, which may relative to the activity of AOSD disease (19). Besides, AOSD patients are often accompanied by increased LDH. Our finding showed a significant correlation between ferritin, liver enzymes, and LDH, suggesting these indicators interact with each other and are closely related to the body's inflammatory response. As the largest organ in the lymphatic system of the whole body, the spleen is responsible for filtering blood and monitoring blood-borne antigens. When an inflammatory reaction occurs, the dendritic cells and T cells of the spleen process antigen and directly contact the pro-inflammatory cytokines in the blood (20). During the activation process, the metabolism of dendritic cells and T cells changes, from oxygen metabolism to glycolysis for energy (21, 22). Therefore, it is feasible to assess spleen immunometabolism through ^18^F-FDG uptake in systemic inflammation (23, 24). Our study demonstrates there is a significant correlation between splenic ^18^F-FDG uptake and laboratory inflammatory parameters. In addition, ^18^F-FDG uptake of bone marrow was associated with CRP, which may be due to the regulation of bone marrow metabolism induced by inflammatory factors (25), suggesting that spleen ^18^F-FDG uptake may provide more comprehensive information in accessing the inflammatory condition of the whole body than traditional laboratory examinations.

Identification of prognostic factors is extremely vital for the proper treatment of AOSD. ^18^F-FDG PET/CT, currently the most commonly used whole-body molecular imaging technology, can not only discover inflammatory reactions and early changes in a disease that cannot be detected by routine laboratory tests but also has significant potential in predicting disease outcome. Since the spleen plays a crucial role in the innate and adaptive immune response, some scholars have indicated that splenic ^18^F-FDG uptake is related to the prognosis of rheumatic immune disease, melanoma, and other diseases (24, 26). In this study, spleen ^18^F-FDG uptake of the AOSD recurrence group was significantly higher than the non-recurrence group, and metabolic parameters of spleen had higher ROC values than ferritin and systemic score, demonstrating splenic radiological uptake may be a potential indicator for predicting AOSD recurrence. Increased spleen ^18^F-FDG uptake may be an overall reflection of the inflammatory state, or it may be an early sign of severe systemic inflammation that has not been detected by traditional laboratory tests (24). Therefore, closer monitoring is required and necessary for AOSD patients with high spleen ^18^F-FDG uptake.

Previous studies revealed that ^18^F-FDG PET/CT could help differentiate AOSD from other febrile autoimmune diseases, and ^18^F-FDG PET/CT may evaluate the therapeutic effect of AOSD (8, 27). Our study further extended the application of ^18^F-FDG PET/CT in assessing AOSD disease activity and predicting of recurrence.

Our study has several limitations. Firstly, it is a single-institution retrospective observational study, with bias in patient selection and analysis. Secondly, only AOSD patients were included. Other differential diagnoses, such as cancer or infection, may present different splenic glucose metabolism patterns and changes.

## Conclusion

The glucose metabolism of the liver, spleen, and bone marrow of AOSD patients were correlated with laboratory inflammatory indicators and systemic score, suggesting that ^18^F-FDG PET/CT could be applied to evaluate disease activity. Moreover, spleen ^18^F-FDG uptake may be a potential biomarker for predicting clinical prognosis of AOSD patients.

## Data Availability Statement

The original contributions presented in the study are included in the article/Supplementary Material, further inquiries can be directed to the corresponding author/s.

## Ethics Statement

The studies involving human participants were reviewed and approved by Medical Ethical Committee of Second Xiangya Hospital. Written informed consent to participate in this study was provided by the participants' legal guardian/next of kin. Written informed consent was obtained from the individual(s) for the publication of any potentially identifiable images or data included in this article.

## Author Contributions

XL: conceptualization, methodology, formal analysis, and writing–original draft. CD: data curation and validation. XM: review and editing, supervision, and writing–review and editing. YW: conceptualization, methodology, supervision, and writing–review and editing. All authors contributed to the article and approved the submitted version.

## Conflict of Interest

The authors declare that the research was conducted in the absence of any commercial or financial relationships that could be construed as a potential conflict of interest.
