# Supplementary material for: 18F-FDG PET/CT Associates With Disease Activity and Clinical Recurrence of AOSD Patients
Source: Front Med (Lausanne). 2021 May 11;8:668323. doi: 10.3389/fmed.2021.668323 (PMC8144305; doi:10.3389/fmed.2021.668323)
Supplement: Supplementary file 1 [file Table_1.DOCX]

**Supplement Table 1** Correlations between quantitative PET measurements and laboratory data

| Variable |  | Liver SUVmax | Liver SUVmean | SlpleenSUVmax | SlpleenSUVmean | Bone marrowSUVmax | Bone marrow SUVmean | lymph node SUVmax | SLR  max | BLR  max | LyLR  max | SLR  mean | BLR  mean |
| --- | --- | --- | --- | --- | --- | --- | --- | --- | --- | --- | --- | --- | --- |
| WBC (/μL) | *r* | 0.029 | -0.182 | -0.058 | -0.059 | 0.134 | 0.085 | -0.222 | -0.106 | 0.154 | -0.249 | 0.017 | 0.213 |
|  | *P* | 0.853 | 0.232 | 0.703 | 0.702 | 0.379 | 0.579 | 0.143 | 0.489 | 0.311 | 0.099 | 0.912 | 0.160 |
| Neutrophil count (/μL) | *r* | 0.091 | -0.139 | -0.047 | -0.081 | 0.190 | 0.130 | -0.198 | -0.133 | 0.157 | -0.237 | -0.024 | 0.230 |
|  | *P* | 0.553 | 0.361 | 0.757 | 0.596 | 0.210 | 0.395 | 0.191 | 0.382 | 0.303 | 0.117 | 0.875 | 0.129 |
| Lymphocyte count (/μL) | *r* | -0.262 | -0.151 | -0.035 | 0.138 | -0.145 | -0.101 | -0.185 | 0.144 | 0.099 | -0.120 | 0.194 | 0.008 |
|  | *P* | 0.083 | 0.323 | 0.821 | 0.365 | 0.343 | 0.507 | 0.224 | 0.344 | 0.518 | 0.433 | 0.202 | 0.958 |
| Monocyte count (/μL) | *r* | -0.003 | 0.095 | -0.135 | -0.077 | -0.218 | -0.184 | -0.214 | -0.155 | -0.178 | -0.236 | -0.173 | -0.227 |
|  | *P* | 0.985 | 0.536 | 0.375 | 0.615 | 0.151 | 0.227 | 0.158 | 0.310 | 0.242 | 0.118 | 0.255 | 0.134 |
| ESR(mm/h) | *r* | 0.021 | -0.071 | -0.026 | -0.094 | 0.229 | 0.213 | -0.009 | -0.081 | 0.131 | -0.021 | -0.067 | 0.247 |
|  | *P* | 0.906 | 0.687 | 0.881 | 0.590 | 0.185 | 0.219 | 0.959 | 0.644 | 0.454 | 0.903 | 0.701 | 0.152 |
| CRP (mg/L) | *r* | -0.087 | -0.218 | 0.100 | 0.050 | .395* | 0.295 | 0.005 | 0.136 | .437** | 0.002 | 0.183 | 0.469** |
|  | *P* | 0.612 | 0.201 | 0.563 | 0.774 | 0.017 | 0.080 | 0.979 | 0.430 | 0.008 | 0.990 | 0.284 | 0.004 |
| ALT (IU/L) | *r* | -0.020 | 0.318 | 0.059 | 0.247 | -0.048 | -0.033 | 0.122 | 0.049 | -0.017 | 0.114 | 0.051 | -0.202 |
|  | *P* | 0.904 | 0.051 | 0.724 | 0.134 | 0.777 | 0.844 | 0.464 | 0.772 | 0.918 | 0.495 | 0.761 | 0.224 |
| AST (IU/L) | *r* | -0.173 | 0.068 | 0.223 | .349* | -0.022 | 0.032 | 0.165 | .413** | 0.172 | 0.248 | .353* | -0.029 |
|  | *P* | 0.300 | 0.687 | 0.179 | 0.032 | 0.898 | 0.851 | 0.322 | 0.010 | 0.302 | 0.133 | 0.030 | 0.864 |
| LDH (IU/L) | *r* | -0.051 | 0.061 | .405* | .539** | 0.036 | 0.019 | 0.244 | .481** | 0.109 | 0.309 | .572** | -0.044 |
|  | *P* | 0.778 | 0.735 | 0.019 | 0.001 | 0.844 | 0.916 | 0.171 | 0.005 | 0.546 | 0.080 | 0.001 | 0.809 |
| Ferritin (mg/ml) | *r* | 0.018 | -0.098 | 0.317 | 0.347 | -0.177 | -0.061 | 0.274 | 0.321 | -0.155 | 0.266 | .586*** | -0.039 |
|  | *P* | 0.924 | 0.595 | 0.077 | 0.052 | 0.331 | 0.739 | 0.129 | 0.074 | 0.398 | 0.141 | 0.001 | 0.831 |
| systemic score | *r* | -0.207 | -0.233 | 0.183 | 0.251 | -0.034 | -0.059 | 0.222 | 0.326* | 0.114 | .295* | .424** | 0.041 |
|  | *P* | 0.172 | 0.124 | 0.228 | 0.096 | 0.823 | 0.701 | 0.143 | 0.029 | 0.456 | 0.049 | 0.004 | 0.788 |

WBC= white blood cell; ESR= erythrocyte sedimentation rate; CRP= C-reactive protein; AST= aspartate aminotransferase; ALT= alanine aminotransferase, LDH=lactate dehydrogenase

**Supplement Table 2** Correlations between laboratory inflammatory parameters

| Variable |  | ESR | CRP | Ferritin | ALT | AST | LDH | systemic score |
| --- | --- | --- | --- | --- | --- | --- | --- | --- |
| ESR | r | / | .430* | -0.150 | -0.290 | -.425* | -.450* | -0.049 |
|  | p | / | 0.013 | 0.473 | 0.113 | 0.017 | 0.021 | 0.785 |
| CRP | r | .430* | / | -0.050 | -0.057 | -0.133 | -0.136 | 0.282 |
|  | p | 0.013 | / | 0.813 | 0.755 | 0.467 | 0.490 | 0.101 |
| Ferritin | r | -0.150 | -0.050 | / | -0.155 | .457* | .545** | 0.335 |
|  | p | 0.473 | 0.813 | / | 0.439 | 0.017 | 0.006 | 0.061 |
| ALT | r | -0.290 | -0.057 | -0.155 | / | .457** | .349* | 0.095 |
|  | p | 0.113 | 0.755 | 0.439 | / | 0.004 | 0.050 | 0.569 |
| AST | r | -.425* | -0.133 | .457* | .457** | / | .861** | .329* |
|  | p | 0.017 | 0.467 | 0.017 | 0.004 | / | 0.000 | 0.044 |
| LDH | r | -.450* | -0.136 | .545** | .349* | .861** | / | 0.276 |
|  | p | 0.021 | 0.490 | 0.006 | 0.050 | 0.000 | / | 0.120 |
| Systemic score | r | -0.049 | 0.282 | 0.335 | 0.095 | .329* | 0.276 | / |
|  | p | 0.785 | 0.101 | 0.061 | 0.569 | 0.044 | 0.120 | / |

ESR= erythrocyte sedimentation rate; CRP= C-reactive protein; AST= aspartate aminotransferase; ALT= alanine aminotransferase, LDH=lactate dehydrogenase

**Supplement Table 3** Comparison of metabolic parameters between AOSD treatment group and untreated group

| Variable | Treatment group | Untreated group | *P* |
| --- | --- | --- | --- |
| Liver SUVmax | 3.59±1.09 | 3.37±0.72 | 0.465 |
| Liver SUVmean | 1.57±0.32 | 1.56±0.38 | 0.983 |
| Spleen SUVmax | 4.78±1.56 | 4.90±1.81 | 0.804 |
| Spleen SUVmean | 2.66±0.91 | 2.87±1.26 | 0.518 |
| Bone SUVmax | 5.72±1.98 | 5.28±1.55 | 0.439 |
| Bone SUVmean | 2.46±0.90 | 2.25±0.70 | 0.408 |
| Lymph node SUVmax | 9.42±6.57 | 11.82±9.24 | 0.314 |
| SLRmax | 1.39±0.50 | 1.47±0.47 | 0.591 |
| BLRmax | 1.66±0.57 | 1.60±0.51 | 0.721 |
| LLRmax | 2.80±2.31 | 3.44±2.04 | 0.347 |
| SLRmean | 1.72±0.52 | 1.84±0.57 | 0.484 |
| BLRmean | 1.59±0.50 | 1.49±0.50 | 0.489 |
